# Development and Characterization of LL37 Antimicrobial-Peptide-Loaded Chitosan Nanoparticles: An Antimicrobial Sustained Release System

**DOI:** 10.3390/polym17131884

**Published:** 2025-07-07

**Authors:** Fazilet Canatan Ergün, Meltem Demirel Kars, Gökhan Kars

**Affiliations:** 1 Department of Biomedical Engineering, Faculty of Engineering, Necmettin Erbakan University, Konya 42140, Turkey; 2 Department of Molecular Biology and Genetics, Faculty of Science, Necmettin Erbakan University, Konya 42140, Turkey; gkars@erbakan.edu.tr

**Keywords:** chitosan, LL37, nanoparticle, ionic gelation, drug release kinetics, biocompatibility, antibacterial

## Abstract

CSNPs synthesized via the ionic gelation method have emerged as a promising nanoplatform in diverse fields such as pharmaceuticals, nanotechnology, and polymer science due to their biocompatibility, ease of fabrication, and tunable properties. This study focuses on the development and characterization of LL37-loaded CSNPs, designed to enhance antibacterial efficacy while maintaining biocompatibility. This study pioneers a systematic loading optimization approach by evaluating the encapsulation efficiency (%EE) of antimicrobial peptide LL37 across multiple concentrations (7.5, 15, and 30 µg/mL), thereby identifying the formulation that maximizes peptide incorporation while preserving controlled release characteristics. The multi-concentration analysis establishes a new methodological benchmark for peptide delivery system development. To achieve this, CSNPs were optimized for size and stability by adjusting parameters such as the chitosan concentration, pH, and stabilizer. LL37, a potent antimicrobial peptide, was successfully encapsulated into CSNPs at concentrations of 7.5, 15, and 30 µg/mL, yielding formulations with favorable physicochemical properties. Dynamic light scattering (DLS) and Zeta sizer analyses revealed that blank CSNPs exhibited an average particle size of 180.40 ± 2.16 nm, a zeta potential (ZP) of +40.57 ± 1.82 mV, and a polydispersity index (PDI) of 0.289. In contrast, 15-LL37-CSNPs demonstrated an increased size of 210.9 ± 2.59 nm with an enhanced zeta potential of +51.21 ± 0.93 mV, indicating an improved stability and interaction potential. Field emission scanning electron microscopy (FE-SEM) analyses exhibited the round shaped morphology of nanoparticles. The release profile of LL37 exhibited a concentration-dependent rate and showed the best fit with the first-order kinetic model. Cytocompatibility assessments using the XTT assay confirmed that both blank and LL37-loaded CSNPs did not exhibit cytotoxicity on keratinocyte cells across a range of concentrations (150 µg/mL to 0.29 µg/mL). Notably, LL37-loaded CSNPs demonstrated significant antibacterial activity against *E. coli* and *S. aureus*, with the 15-LL37-CSNP formulation exhibiting superior efficacy. Overall, these findings highlight the potential of LL37-CSNPs as a versatile antibacterial delivery system with applications in drug delivery, wound healing, and tissue engineering.

## 1. Introduction

Nanotechnology is an emerging field that focuses on the manipulation and application of materials at the nanometer (nm) scale. One of the fundamental building blocks of nanotechnology is nanoparticles, which have recently gained significant attention across diverse fields, including pharmaceuticals, therapeutic innovations, nanotechnology, and polymer science [1]. IUPAC refers to particles with all external dimensions in the range of approximately 1–100 nanometers as nanoparticles. In practical applications, particularly in the biomedical, pharmaceutical, and materials science fields, the term ‘nanoparticles’ is frequently used for chitosan particles in the size range of 100–500 nm, despite exceeding the IUPAC-defined limit. This broader usage is often justified by the soft and hydrophilic nature of polymeric materials, which tend to swell in aqueous media [2]. Due to their nanoscale dimensions and high surface-area-to-volume ratio, nanoparticles exhibit unique physical and chemical properties, making them ideal for various biomedical applications. They can be synthesized using both natural and synthetic polymers; however, natural polymers are often preferred due to their lightweight nature, ease of preparation, and avoidance of organic solvents and high shear forces during synthesis.

Polymers are composed of multiple repeating monomer units. Numerous natural polymers, such as proteins (e.g., collagen, elastin, keratin, and silk fibroin) and polysaccharides (e.g., alginate, chitosan, hyaluronic acid, and cellulose), as well as synthetic polymers (e.g., polylactic acid (PLA), polyvinyl alcohol (PVA), polycaprolactone (PCL), poly (lactic-co-glycolic acid) (PLGA), and polyethylene glycol (PEG)), are widely used in medical applications [3]. It has been demonstrated that natural biopolymers such as chitosan, collagen, and hyaluronic acid (HA) support wound healing, particularly by stimulating anti-inflammatory responses in chronic wounds [4].

Chitosan (CS), a versatile natural biopolymer abundantly available in nature, is widely used for nanoparticle preparation due to its desirable properties, such as low cost, biodegradability, biocompatibility, and non-toxicity [1]. Chitosan is a linear natural polysaccharide derived from the partial deacetylation of chitin under alkaline conditions [5]. Structurally, it consists of β−1,4-linked glucosamine and N-acetylglucosamine residues, offering numerous valuable attributes, including biocompatibility, biodegradability, low toxicity, immune stimulation, mucoadhesive properties, the proton sponge effect, antimicrobial activity, and enhanced bioavailability [6]. CS was selected as the carrier matrix because it uniquely combines biodegradability, biocompatibility, and intrinsic antimicrobial activity in a single biopolymer—properties that are rarely co-localized in alternative materials. Unlike neutral or anionic polysaccharides, chitosan’s primary amine groups become protonated under mildly acidic to physiological pH, imparting a cationic surface charge that promotes electrostatic interactions with negatively charged bacterial membranes and mucosal tissues, thereby enhancing both antibacterial efficacy and mucoadhesion. Moreover, CS has been classified as “Generally Recognized As Safe” by the U.S. Food and Drug Administration, reflecting its minimal cytotoxicity and favorable safety profile in both oral and topical applications [7]. Recent comprehensive reviews confirm that CS exhibits a superior antioxidant capacity and enzyme inhibitory effects compared to other biopolymers such as alginate or cellulose derivatives, and its ease of chemical modification enables the precise tuning of release kinetics in drug delivery systems [8]. Taken together, these distinctive attributes make CS the optimal choice for developing a sustained release antimicrobial peptide delivery platform.

Chitosan nanoparticles (CSNPs) are commonly synthesized using the ionic gelation method, which is based on electrostatic interactions between oppositely charged ions [9]. Among various ionic gelation approaches, the use of tripolyphosphate (TPP) as a crosslinking agent is the most popular due to its ease of application, low toxicity, and scalability. The size of nanoparticles plays a critical role in determining their biological performance, including biodistribution, stability, drug loading capacity, drug release profile, and toxicity [10]. Moreover, nanoparticle size significantly influences intracellular uptake, transport, and movement in biological fluids [11].

Recent studies have highlighted the promising antimicrobial potential of CSNPs. For instance, Al-Zahrani et al. [12] demonstrated that CSNPs exhibited significant antibacterial activity. Additionally, Pan et al. [13] reported that TPP-loaded CSNPs showed enhanced inhibitory effects against *Escherichia coli* and *Staphylococcus aureus* compared to TPP-free CSNPs. However, the practical use of CSNPs in industrial applications can be challenging due to the high concentrations required, which may not be cost-effective and could induce undesirable changes in the targeted products. To address these limitations, the incorporation of effective antibacterial agents into CSNP formulations has been proposed as a strategy to enhance their antimicrobial efficiency while reducing the required dosage [12].

Antimicrobial Susceptibility Testing (AST) is essential for evaluating the effectiveness of antimicrobial agents, guiding treatment, tracking resistance, and developing new formulations. Integrating AST into the study of delivery systems like chitosan nanoparticles (CSNPs) is vital for understanding their therapeutic potential. One of the most widely used AST methods is the microdilution test, which determines the minimum inhibitory concentration (MIC) through serial dilutions [14]. It offers a high precision and reproducibility, and is well-suited for assessing nanoparticle-based formulations under physiological like conditions. Its scalability and compatibility with high-throughput formats make it valuable in both research and diagnostics. The gradient diffusion method is another common approach, using antibiotic-impregnated strips on agar to provide semi-quantitative MIC values [15]. Innovative techniques like di-electrophoresis, based on the movement of bacterial cells in electric fields, and optoelectronic sensors, detecting growth-related signal change, offer rapid, label-free, and sensitive alternatives for AST [16,17]. Together, these methods form a comprehensive framework for evaluating antimicrobial activity and are particularly important for assessing the potency and mechanism of advanced delivery systems.

Antimicrobial peptides (AMPs) have emerged as promising alternatives in the fight against bacterial infections [18]. AMPs are typically positively charged molecules that interact with the negatively charged bacterial membranes, leading to membrane disruption and bacterial cell death [19]. Among AMPs, LL37 is a physiologically relevant, positively charged, amphipathic, α-helical peptide with potent antibacterial properties at a pH of 6 [20]. LL37 exhibits broad-spectrum antimicrobial activity by disrupting the bacterial membrane integrity [21]. However, despite its effectiveness, LL37 has several limitations, such as poor stability in biological environments, susceptibility to proteolytic degradation, weak interactions with host macromolecules, and potential toxicity concerns [22]. Encapsulation within suitable delivery systems, such as CSNPs, has been proposed to overcome these challenges and improve the stability and bioactivity of LL37 [23].

In this context, the development of an effective delivery system by loading LL37 onto CSNPs with optimized properties represents a promising strategy for enhancing its antimicrobial potential. Various characterization techniques, including DLS for determining the particle size and zeta potential, and Scanning Electron Microscopy (SEM) for morphological analysis, are commonly employed to evaluate the physicochemical properties of CSNPs [1]. Chitosan-based nanoparticles have shown reduced toxicity when used in mucoadhesive formulations [24], further supporting their potential application in biomedical fields such as drug and gene delivery, tissue engineering, bone regeneration, and wound healing [25].

The primary objective of this study is to optimize the formulation of CSNPs by evaluating critical parameters such as the chitosan concentration, TPP concentration, chitosan-to-TPP volume ratio, and the effects of additives such as NaCl and Tween 80. The optimized CSNP formulation was then used for the encapsulation of LL37, followed by an assessment of its encapsulation efficiency, release kinetics, and antibacterial activity. Additionally, the biocompatibility of the blank and LL37-loaded CSNPs was evaluated and compared to suggest their potential use for biomedical applications.

## 2. Materials and Methods

### 2.1. Chemicals and Materials

CS with a low molecular weight and a deacetylation degree of 95% or higher (Sigma-Aldrich,, St. Louis, MO, USA), TPP (Sigma-Aldrich, St. Louis, MO, USA), ultra-pure water, analytical grade acetic acid (AA, glacial, 99%, 5%), Tween 80, NaCl, sodium hydroxide (NaOH), hydrochloric acid (HCl), Dulbecco’s Modified Eagle Medium (DMEM) (Gibco, Grand Island, NY, USA), fetal bovine serum (FBS), gentamicin (Sigma-Aldrich), LL37 peptide (LLGDFFRKSKEKIGKEFKRIVQRIKDFLRNLVPRTE) (Chinapeptides, Shanghai, China), Micro BCA Assay Kit (Thermo Fisher Scientific, Waltham, MA, USA), and XTT (3-(4,5-dimethylthiazol-2-yl)-2,5-diphenyltetrazolium bromide assay, Sartorius, Göttingen, Germany) were purchased, and all chemicals and reagents were used as received.

### 2.2. Method

#### 2.2.1. Development of CSNPs

**Figure 1:**
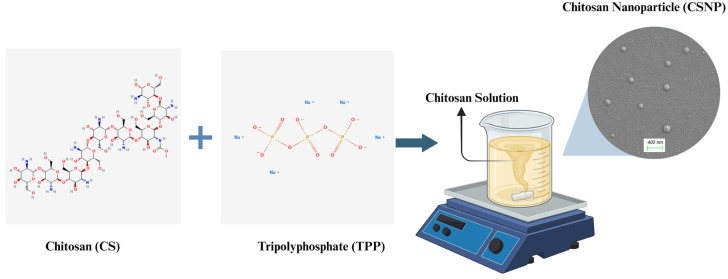
Schematic representation of CSNP production.

In the initial stage of the study, solutions containing varying concentrations of CS and sodium TPP were prepared to optimize the nanoparticle formulation. The optimization process aimed to identify the optimal parameters for CSNP production by evaluating different CS:TPP ratios and process conditions. The parameters utilized for CSNP production are summarized in Table 1, providing a comprehensive overview of the experimental design. To facilitate a better understanding of the CSNP production process, a schematic representation is provided in Figure 1, illustrating the key steps involved in the preparation of chitosan nanoparticles via the ionic gelation method.

CSNPs (1–4) were produced using variable process medium, and the resulting CSNPs were compared. Firstly, the CS (1% *v*/*v*) was dissolved in acetic acid solution, and TPP was dissolved in ultra-pure water for 24 h. Under room conditions, the volume ratio of CS:TPP (2:1) was added dropwise at a flow rate of 0.25 mL/min and mixed on a magnetic stirrer. The CSNP solution was mixed in a homogenizer (7000 rpm for 2 min) prior to centrifugation. Following homogenization, the suspension was centrifuged at 14,000 rpm for 40 min and the pellet was washed with deionized water. This washing step was repeated three times to effectively remove residual acetic acid and excess unbound CS from the surface of the nanoparticles. The washed nanoparticles were then resuspended in PBS (pH 6.8), ensuring a physiologically compatible dispersion for downstream analysis. For filtration, the solution was filtered sequentially with 0.45 µm and 0.22 µm syringe filters.

#### 2.2.2. Development of LL37-Loaded CSNPs

The optimal nanoparticle (NP) production parameters were determined based on a comparative analysis of the physicochemical characteristics of four CSNP formulations (CSNP-1 to CSNP-4). The CSNP-1 formulation was identified as the most suitable, and its process conditions were selected for further studies. Following the optimization, the production parameters of blank CSNP-1 were utilized for the encapsulation of LL-37 at different concentrations (30, 15, and 7.5 µg/mL). The resulting LL37-loaded CSNP groups were designated as 30-LL37-CSNP, 15-LL37-CSNP, and 7.5-LL37-CSNP, respectively.

To ensure removal of residual acetic acid and unbound chitosan, the LL37-loaded chitosan nanoparticles (CSNPs) underwent thorough purification following synthesis. After preparation via ionic gelation, the nanoparticle suspension was centrifuged and washed three times with deionized water to eliminate free chitosan and any remaining acid. The final nanoparticle pellet was then resuspended in PBS (pH 6.8), providing a neutral, physiologically compatible medium for further analysis. Importantly, during the entire encapsulation process, the pH was carefully monitored and maintained above 6.0 to preserve the structural integrity of LL37 and avoid acid-induced conformational changes. The supernatants were collected and stored at −20 °C for further Micro BCA analysis, while the obtained pellets were lyophilized for long-term storage and subsequent characterization.

#### 2.2.3. Physicochemical Characterization of CSNPs

The particle size, PDI, and ZP of CSNPs were measured using a DLS system (Zetasizer NanoPlus-3, Micromeritics, Norcross, GA, USA). The mean particle size was determined based on the average of three independent measurements, with each measurement comprising 50 readings. The morphological characteristics of chitosan nanoparticles (CSNPs) were analyzed using a FESEM (GeminiSEM 500, ZEISS, Oberkochen, Germany). The samples were examined under an accelerating voltage of 2 kV to ensure high-resolution imaging.

#### 2.2.4. Encapsulation Efficiency of LL-37 and In Vitro Release Kinetics

The encapsulation efficiency (EE) of LL37-loaded chitosan nanoparticles (30-LL37-CSNP, 15-LL37-CSNP, and 7.5-LL37-CSNP) was determined using the protein detection assay Micro BCA (Thermo Scientific) and calculated based on Equation (1).

(1) $$EE\left(\%\right)=\frac{TotalLL37concentration-FreeLL37concentration}{TotalLL37concentration}\times 100$$

A calibration curve for the Micro BCA assay was established using bovine serum albumin (BSA) as the standard [26,27,28], within a concentration range of 0 to 40 μg/mL, yielding a coefficient of determination R^2^ of 0.9986. All measurements were conducted in triplicate to ensure reproducibility. The formulation that exhibited EE% more than 80 percent was used to conduct the in vitro LL37 release measurements. The in vitro release profile of LL37 from CSNP formulations was evaluated by suspending the LL37-CSNPs in phosphate-buffered saline (PBS, pH 6.8) at 37 °C. Supernatants were collected at 24 h intervals over a period of 24 days, and LL37 release was quantified using a calibration curve constructed with the albumin standard in the Micro BCA assay. The calibration curve included standard concentrations of 40, 20, 10, 5, 1, and 0 μg/mL. The encapsulation efficiency was determined based on the LL37 content obtained from this calibration curve.

To characterize the release kinetics of LL37 from CSNPs, the experimental data were fitted to several mathematical models, including zero-order, first-order, Higuchi, Korsmeyer–Peppas, and Hixson–Crowell models. These models provide insights into the drug release mechanism and kinetics. Zero-order kinetics defines the constant release of a drug from a formulation, which is independent of time or concentration; zero-order release is expressed by Equation (2), where *C*_0_ is the initial drug concentration, *C_t_* is the amount of drug released at time ‘t’, and *K*_0_ is the zero-order rate constant. Data fitting was performed by plotting the cumulative percentage of drug release against time.

(2) $$C_{0}-C_{t}=K_{0}t$$

First-order kinetics means the drug release occurs exponentially as defined by Equation (3), where *C*_0_ is the initial drug concentration, t is time, and *K_t_* is the first-order rate constant. The obtained data were plotted as the cumulative logarithmic percentage of the remaining drug over time.

(3) $$LogC=LogC_{0}-\left(\frac{K_{t}}{2.303}\right)$$

The Korsmeyer–Peppas semi-empirical model predicts drug release mechanisms from polymeric systems and follows Equation (4), where *C_t_/C_∞_* represents the fraction (ratio) of drug release at time *t*, and *K* is the rate constant. The logarithm of the cumulative drug release percentage was plotted against the logarithm of time.

(4) $$\frac{C_{t}}{C_{\infty }}={Kt}^{n}$$

The Higuchi model describes drug release from matrix-based systems, where the drug diffusion follows Fick’s law. The release kinetics are represented by Equation (5), where *C_t_* represents the amount of drug released at time *t*, and *K_H_* is the Higuchi rate constant. The cumulative percentage of drug release was plotted against the square root of time to evaluate the model’s applicability.

(5) $$C_{t}=K_{H}\;\times \sqrt{t}$$

The Hixson–Crowell model describes drug release as being dependent on the surface area and volume of the particles. The mathematical expression for this model is presented as Equation (6), where *K_H_* is the rate constant, *C*_0_ is the initial drug concentration, and *C_t_* represents the drug release at time t. The data were fitted by plotting the cube root of the remaining drug percentage against time [29].

*C_0_*^1/3^ − *C_t_*^1/3^ = *K_H_ C_t_* (6)

The results were compared across these kinetic models to identify the best fit and to elucidate the potential release mechanisms governing LL37 delivery from the CSNP formulations.

Kinetic models involve mathematical equations that describe the drug release mechanism, and regression analysis is used to fit these models to experimental data. Regression is applied to assess the degree of agreement between the mathematical models and the observed data. Through regression analysis, various statistical metrics such as the coefficient of determination (R^2^), Root Mean Square Error (RMSE), Residual Sum of Squares (RSS), Akaike Information Criterion (AIC), and Bayesian Information Criterion (BIC) are calculated for each kinetic model. These metrics are compared to determine the best-fitting model. R^2^ indicates how well the model explains the variability in the data and ranges between 0 and 1. A value of 0 means the model does not explain the data, while a value of 1 indicates a perfect fit. RMSE measures the predictive accuracy of the model; lower RMSE values suggest a better fit. RSS represents the total amount of error in the model’s predictions, with smaller values indicating closer alignment with the actual data. AIC evaluates both the goodness of fit and the complexity of the model; among competing models, a lower AIC value generally indicates a better model. BIC is like AIC but includes a stronger penalty term for model complexity. Like AIC, BIC is used for model comparison, and lower BIC values indicate a better model fit [30].

#### 2.2.5. Evaluation of In Vitro Biocompatibility

The in vitro biocompatibility of CSNP formulations was assessed by evaluating their effects on the proliferation of keratinocyte cells. Initially, keratinocyte cells were cultured in DMEM supplemented with 10% FBS and antibiotics to ensure optimal growth conditions. To investigate any cytotoxic effects of CSNPs, the cells were exposed to ten different concentrations of CSNPs, ranging from 150 µg/mL to 0.29 µg/mL. Control groups were included, consisting of wells designated as cell control (without CSNPs but with cells) and medium control (without cells and CSNPs). Actively proliferating cells, excluding the medium control wells, were seeded in 96-well culture plates at a density of 5000 cells per well in a volume of 50 μL of complete medium. The plates were incubated at 37 °C in a humidified 5% CO_2_ incubator for 24, 48, and 72 h to allow cell proliferation. Following the incubation period, the mitochondrial activity of viable cells was assessed using the 3-(4,5-dimethylthiazol-2-yl)-2,5-diphenyl tetrazolium bromide (XTT) assay (Sartorius). The XTT reagent was added to each well and incubated for an additional 4 h to enable the enzymatic conversion of the tetrazolium salt to a formazan product by metabolically active cells. Absorbance was measured at 490 nm using an ELISA plate reader to determine the percentage of viable cells. The cell viability for each test condition was calculated relative to the control group, where the untreated control cells were considered to represent 100% viability. The effects of both blank and LL37-loaded CSNP formulations on cell proliferation were analyzed and compared by calculating the relative cell viability percentage. This analysis provided insights into the biocompatibility of the CSNP formulations and their potential impact on keratinocyte cell proliferation.

#### 2.2.6. Evaluation of Antibacterial Activity

The antibacterial activity of CSNP formulations was assessed against both Gram-positive *Staphylococcus aureus* (ATCC 29213) and Gram-negative *Escherichia coli* (ATCC 25922) bacterial strains. Samples from the CSNP groups were prepared by applying 25 µL of each sample (150 µg/mL) onto disk-shaped filter paper. The disks were allowed to air-dry to ensure uniform distribution of the formulation.

For each bacterial strain, three colonies were selected from cultured plates and suspended in 4–5 mL of Mueller–Hinton Broth (MHB). The bacterial suspensions were adjusted to a density of 0.5 McFarland standard, corresponding to approximately 5 × 10^6^ CFU/mL. A 96-U-bottomed-well microplate was used for the assay, with a final volume of 100 µL per well. The bacterial suspension was inoculated into each well to achieve the desired cell density. The prepared CSNP-coated filter paper discs were placed into the inoculated wells in triplicate to ensure reproducibility. The microplate was incubated at 37 °C for 16 h under appropriate conditions to allow bacterial growth and CSNP interaction. At the end of the incubation period, the filter paper discs were removed from the wells, and bacterial growth was evaluated by measuring the absorbance at 600 nm using an ELISA microplate reader (Multiskan Reader, Thermo Fisher Scientific, Waltham, MA, USA). The absorbance values were used to compare the bacterial viability with control group and to assess bacterial viability in the CSNP-treated wells. This method provides a quantitative evaluation of the antibacterial properties of CSNPs against clinically relevant bacterial strains, offering insights into their potential applications in antibacterial therapies.

## 3. Results and Discussion

### 3.1. Physicochemical Characteristics of CSNPs and LL37-CSNPs

The present study highlights the impact of formulation and process parameters on the physicochemical properties of CSNPs. The findings demonstrate that appropriate CS:TPP ratios, controlled pH conditions, and optimized stirring rates contribute to achieving nanoparticles with desirable size, distribution, and stability characteristics. The experimental results provide valuable insights for the development of stable and effective CSNP formulations. According to Table 2, the average nanoparticle size, and PDI and ZP values for CSNP-1 are at the optimum values compared to other groups. The LL37-loaded CSNP was developed using CSNP-1 parameters to encapsulate LL37. CSNP-1 was selected for LL37 loading because it combined the smallest baseline particle diameter with a low PDI and a suitably high zeta potential among all tested chitosan nanoparticle formulations. Particle size and PDI are widely recognized as key quality attributes in nanocarrier design [31], since smaller, more uniform nanoparticles generally exhibit better stability and predictable performance. Crucially, encapsulating a peptide payload typically causes nanoparticle enlargement (as has been observed for chitosan carriers loaded with protein or peptide cargos) [32]. By beginning with CSNP-1’s minimal mean size, we ensured that the inevitable size increase upon LL37 binding would still result in optimally small nanoparticles. In addition, CSNP-1’s narrow size distribution and strong surface charge provided good colloidal stability, further supporting its suitability for loading. In summary, CSNP-1’s unique combination of the smallest initial size, uniformity (low PDI), and robust zeta potential made it the ideal candidate for LL37 encapsulation—it offered the maximum size “buffer” to accommodate the post-loading size increase while preserving desirable nanoparticle properties.

Among the critical parameters influencing the physicochemical properties of CSNPs, the CS:TPP ratio plays a significant role in determining the particle size, distribution, and ZP. Increasing the CS:TPP volume ratio correlates with an increase in nanoparticle size, as a higher volume of CS helps to prevent precipitation and agglomeration. However, an excessive CS proportion can lead to larger particles, as reported by Algharib et al. [11]. Based on these findings, a CS:TPP ratio of 2:1 was selected in the present study to ensure optimal nanoparticle stability and size distribution. The primary role of NaCl in the CS and TPP solution mixtures was to enhance the control over nanoparticle formation by reducing the rigidity and electrostatic repulsion of CS chains. This results in a more compact particle formation, as corroborated by previous studies [33,34]. Additionally, an optimal concentration of NaCl is crucial for achieving colloidal stability and a uniform particle size distribution, whereas excessively high concentrations can impede the CS-TPP interaction. Studies have also shown that ionized salts weaken the electrostatic interactions between CS and TPP, further influencing the nanoparticle formation [35].

The CS concentration is a pivotal factor in determining the physicochemical characteristics of CSNPs. Higher CS concentrations lead to increased intermolecular interactions and crosslinking with TPP, resulting in the formation of larger particles. This size increment is attributed to the presence of hydroxyl (–OH) groups in CS, which facilitate intermolecular hydrogen bonding, and protonated amino (–NH_3_^+^) groups, which contribute to electrostatic repulsion [36]. Morphological variations in CSNPs have been observed with changing CS concentrations [11]. Similarly, increasing the TPP concentration promotes greater CS-TPP complex formation, potentially leading to particle aggregation, which is a well-documented limitation of CSNP systems [37].

The incorporation of Tween 80 has been identified as an essential factor in preventing nanoparticle aggregation. Studies have demonstrated that the absence of Tween 80 results in failed nanoparticle formation, while its presence enhances stability. However, increasing the concentration of Tween 80 can contribute to larger particle sizes. For instance, CSNP sizes were reported as 148.8 ± 1.1 nm (PDI = 0.066) at 0.5% Tween 80 and 177.0 ± 3.2 nm (PDI = 0.090) at 1% Tween 80 [1,38]. These variations are further influenced by factors such as the molecular weight and degree of deacetylation of CS [39,40,41]. CSNP-1 was indeed prepared with Tween-80 as specified in the Methods and Table 1. We explicitly optimized all formulations for the smallest mean size, optimal PDI (narrowest size distribution), and optimal zeta-potential (for colloidal stability). By these criteria, CSNP-1 (with Tween-80) proved superior: it produced the smallest, most monodisperse particles with the strongest surface charge. This result is consistent with the known effects of Tween-80 in chitosan nanoparticle synthesis. Tween-80, a non-ionic surfactant, increases chitosan solubility and lowers interfacial tension, which drives the formation of much smaller nanoparticles [8]. Darwish et al. similarly found that a higher Tween-80 content steadily reduced the vesicle size, noting that formulations with the highest Tween-80 gave the smallest particles; they explicitly describe Tween-80 as a stabilizing agent that reduces the surface energy and prevents particle coalescence [42]. In practice, the surfactant forms a steric barrier around each nanoparticle, inhibiting aggregation and yielding a narrow PDI. Consistent with this, formulations containing Tween (or Tween-20, a close analog) show dramatically improved PDI and stability; the capacity to maintain particle integrity by employing Tween is much greater than in surfactant-free systems [43]. In contrast, simple ionic additives like NaCl (used in CSNP-2/4) can only screen the charge and do not provide this steric stabilization, so they do not achieve as small or uniform a particle population. Thus, the inclusion of Tween-80 in CSNP-1 explains its optimized size/PDI/zeta profile, whereas NaCl-containing variants gave larger, broader dispersions. The established literature on chitosan/Tween-80 nanoparticles reports that Tween-80 significantly reduces particle size and PDI by improving solubility and preventing aggregation, in agreement with our findings.

The method of CSNP preparation significantly affects the particle properties, with the dropwise addition of the CS solution to the TPP solution being crucial for controlled nanoparticle formation. The flow rate and mixing time during this process are critical factors influencing particle size and PDI. Studies indicate that higher flow rates of TPP lead to increased particle size and PDI values, which aligns with the findings of Majedi et al. [44]. The pH of the CS and TPP solutions is another essential environmental factor governing CSNP formation. An acidic medium is necessary for facilitating the solubility of CS and ensuring the protonation of amino groups, which promotes effective solvent diffusion and prevents premature aggregation [45]. It has been observed that pH values exceeding 5.5 lead to decreased protonation and increased agglomeration [46].

The magnetic stirring speed during the CSNP synthesis process significantly affects particle formation. Optimal stirring facilitates the uniform dispersion of TPP in the CS solution, promoting efficient crosslinking and size reduction. However, excessive stirring can eliminate repulsive forces, leading to aggregation [36,47]. In a study by Algharib et al. [11], particle sizes of 138.50 ± 0.50 nm at 500 rpm, 67.17 ± 0.77 nm at 1000 rpm, and 105.50 ± 0.50 nm at speeds above 1000 rpm were reported. In this study, a stirring speed of 800 rpm was maintained to achieve the desired nanoparticle size.

The polydispersity index (PDI) is an important parameter used to evaluate particle size distribution in nanoparticle suspensions. A lower PDI indicates a uniform particle size distribution, while higher values reflect polydispersity [48]. In the current study, the PDI values for the blank CSNP (1-2-3-4), 7.5-LL37-CSNP, and 15-LL37-CSNP groups ranged between 0.2 and 0.4, suggesting a homogeneous and narrow particle size distribution (Table 2). Similar findings were reported by Algharib et al. [11], where an increase in CS concentration correlated with increased PDI values due to higher agglomeration tendencies.

**Figure 2:**
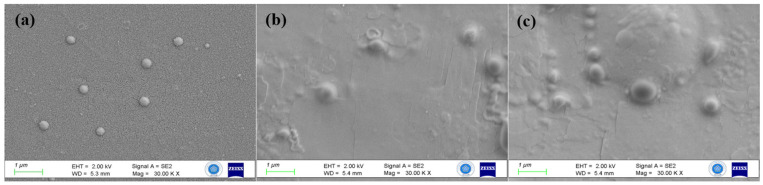
FE-SEM images of (**a**) CSNP-1, (**b**) 7.5-LL37-CSNP, and (**c**) 15-LL37-CSNP.

ZP is a key indicator of nanoparticle stability, with values exceeding ±30 mV considered essential for physical stability [49]. Lazaridou et al. reported ZP values for CSNPs in the range of +37.6 to +40.1 mV [50]. In the current study, the ZP values for CSNP (1-2-3-4), 7.5-LL37-CSNP, and 15-LL37-CSNP were determined as 40.57, 43.68, 46.26, 35.75, 19.26, and 51.21 mV, respectively. These values exceed the stability threshold, indicating excellent colloidal stability and potential for an enhanced cellular uptake due to the interactions with negatively charged cell membranes [11]. The morphological characteristics of CSNPs were analysed using FE-SEM imaging. The formulations containing Tween 80 exhibited a distinctly spherical morphology (Figure 2).

DLS and Zeta sizer analyses revealed that blank CSNPs exhibited an average particle size of 180.40 ± 2.16 nm, a zeta potential of +40.57 ± 1.82 mV, and a polydispersity index (PDI) of 0.289. In contrast, 15-LL37-CSNPs demonstrated an increased size of 210.9 ± 2.59 nm with an enhanced zeta potential of +51.21 ± 0.93 mV, indicating an improved stability and interaction potential.

Figure 2 presents the FE-SEM images that reveal the morphological features of nanoparticle formulations. CSNP-1 shows a smooth spherical morphology, while LL37-loaded nanoparticles (7.5-LL37-CSNP and 15-LL37-CSNP) reveal an increased surface roughness and slight size augmentation. These changes likely reflect the LL37 integration via electrostatic interactions, which is consistent with the literature reporting the peptide-induced surface heterogeneity in CSNP [51]. The preserved spherical form and limited aggregation in CSNP-1 support its selection as the foundational structure for peptide loading. A critical mechanistic insight underpinning our system’s novelty lies in the electrostatic and amphipathic helix-mediated interactions between CSNPs and the LL37 antimicrobial peptide. Chitosan’s primary amine groups, which impart a strong cationic surface charge at physiological pH, engage in electrostatic attraction with the negatively charged residues of LL37, promoting efficient peptide adsorption or encapsulation within the nanoparticle matrix [52,53,54]. Furthermore, LL37 inherently adopts an amphipathic α-helical conformation under physiological conditions, aligning its hydrophobic and charged regions in a manner that supports strong hydrophobic and electrostatic interactions with the chitosan backbone. This structural complementarity facilitates a densely packed peptide–polymer complex, enhancing the encapsulation efficiency and contributing to a controlled release profile.

SEM images indicated that increasing the LL37 concentration was associated with a modest increase in particle size and surface roughness. This may be due to the stronger electrostatic and hydrophobic interactions between the cationic chitosan matrix and the amphipathic LL37 peptide, potentially resulting in denser internal packing or localized surface heterogeneity. Although the images were captured shortly after synthesis, we recognize that the storage duration and physiological exposure could further alter the nanoparticle architecture. These structural dynamics may have meaningful implications in vivo. For instance, surface irregularities may influence cell–nanoparticle interactions, mucosal adhesion, or the ability to penetrate biological barriers. Likewise, changes in particle integrity over time could impact the drug release profile, bioavailability, and, ultimately, therapeutic efficacy. Supporting this, Fahimirad et al. (2021) demonstrated that LL37-loaded chitosan nanoparticles significantly improved wound healing outcomes in a mouse model by enhancing the antibacterial properties and accelerating tissue regeneration, compared to the free LL37 treatment. Their findings attributed this effect to both the structural stability of the CSNPs and the sustained release behaviour of the encapsulated peptide [55]. Based on this evidence, we plan to extend our work through long-term stability studies and in vitro simulations of physiological environments (e.g., 3D dermal models) to better predict the performance of LL37-CSNPs in biomedical applications.

### 3.2. Encapsulation Efficiency

The encapsulation efficiency (%EE) of LL37 within chitosan nanoparticles (CSNPs) was assessed using the Micro BCA protein assay absorbance values at 562 nm. The calculated %EE values, derived from the LL37 standard curve, are summarized in Table 3. Based on the loaded LL37 concentrations, the %EE values for CSNPs loaded with 30 µg/mL, 15 µg/mL, and 7.5 µg/mL of LL37 were determined to be 55.57%, 80.32%, and 97.81%, respectively.

A higher encapsulation efficiency was observed for CSNPs loaded with 15 µg/mL and 7.5 µg/mL of LL37, indicating a more effective incorporation of the peptide at these concentrations. Specifically, the %EE value of 55.57% for CSNPs loaded with 30 µg/mL LL37 suggests that nearly half of the peptide was successfully encapsulated, while the remaining portion remained unencapsulated. In contrast, the encapsulation efficiencies of 80.32% and 97.81% for 15-LL37-CSNP and 7.5-LL37-CSNP formulations, respectively, demonstrate a superior loading efficiency at lower LL37 concentrations. The findings of this study are consistent with the previously reported values in the literature. Rashki et al. [26] reported an encapsulation efficiency of 86.9% for LL37-loaded CSNPs, which aligns closely with the values obtained for the 15-LL37-CSNP formulation in the present study. Similarly, Nair et al. [29] investigated curcumin-loaded chitosan nanoparticles at different CS:curcumin ratios (5:1, 4:1, and 3:1) and reported encapsulation efficiencies of 80.4%, 80.2%, and 88.4%, respectively, supporting the trends observed in the current findings. Furthermore, Piras et al. [56] reported an encapsulation efficiency of approximately 75% for temporin within CSNPs, further corroborating the efficiency of chitosan as a nanoparticle carrier system. These results underscore the effectiveness of chitosan nanoparticles in encapsulating LL37, with lower loading concentrations yielding higher encapsulation efficiencies. The findings highlight the potential of CSNPs as a promising delivery vehicle for antimicrobial peptides such as LL37, offering controlled release and improved bioavailability.

### 3.3. In Vitro LL37 Release from CSNPs

The drug release profile of CSNPs plays a crucial role in their therapeutic efficacy [51]. The release characteristics are influenced by various physicochemical properties such as the particle shape, size, degradation rate, chemical composition, molecular weight, and solubility. Furthermore, interactions between the drug and polymer matrix, as well as potential drug–drug interactions, significantly impact the release behaviour [57].

**Figure 3:**
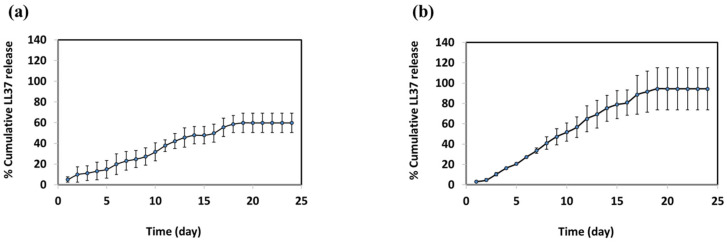
Cumulative release profile of LL37 from (**a**) 7.5-LL37-CSNP and (**b**) 15-LL37-CSNP.

The cumulative release profiles of LL37 from 15-LL37-CSNP and 7.5-LL37-CSNP over a period of 24 days in phosphate-buffered saline (PBS, pH 6.8) at 37 °C are presented in Figure 3. At the end of the 24-day period, the cumulative release of LL37 was determined to be 94.39% for 15-LL37-CSNP and 59.86% for 7.5-LL37-CSNP. The release profile exhibited a linear and sustained release pattern up to day 18, after which the remaining LL37 was gradually released between days 18 and 24. The prolonged and incomplete release of LL37 is likely due to the strong interactions between LL37 and the chitosan polymer, which hinder its diffusion into the surrounding medium. This phenomenon is consistent with the findings of Tığlı and Pulat [58], who reported that 5-fluorouracil was released from chitosan in a sustained manner under in vitro conditions, with the cumulative release ranging between 29.1% and 60.8% after 17 days, depending on pH variations.

A major challenge associated with the free form of LL37 is its inherent instability and susceptibility to rapid degradation. Encapsulation within CSNPs provides protection against environmental degradation, thus enhancing its stability and ensuring a sustained therapeutic action [55,59,60]. However, the slow-release rate observed in this study may be attributed to the rigid and hydrophobic core of the CSNPs, which restricts the diffusion of LL37 from the nanoparticle matrix. As reported by Li et al. [61], upon exposure to an aqueous environment, the polymer gradually degrades, allowing the encapsulated LL37 to diffuse into the medium over time.

To characterize the release kinetics of LL37 from CSNPs, mathematical models were applied to the experimental data, and the best-fitting model was selected based on the highest correlation coefficient (R^2^) and the lowest RMSE, RSS, AIC, and BIC values. As presented in Table 4, the release profiles of both 7.5-LL37-CSNP and 15-LL37-CSNP were best described by the first-order release model. The values in Table 4 indicate that the release rate of LL37 is dependent on the remaining amount of the compound, suggesting a delayed yet sustained release process. In first-order release kinetics, the release rate of the active compound is proportional to the remaining drug amount. Therefore, as the amount of the drug decreases, the release rate also declines. In the study conducted by Sun et al., 5-FU-loaded chitosan nanoparticles (5-FU-CN) were prepared, and their sustained release behaviour was investigated through an in vitro release and initial in vivo pharmacokinetic studies. The authors reported that 5-FU-CN followed a first-order kinetic model [62]. Similarly, in the study by Nasri et al., the best-fit kinetic model for microparticles (MPs) was evaluated by determining the highest R^2^ value. Overall, the release kinetics of biopeptides from chitosan MPs were found to fit well with the first-order (0.992–0.995) and Korsmeyer–Peppas (0.995–0.996) models [63]. These findings are consistent with the results of the present study.

The findings of the present study confirm a logarithmic release profile in which the amount of LL37 released decreases over time as the amount of LL37 remaining within the capsule is reduced, indicating a prolonged release process extending over 24 days. The strong interaction between LL37 and the CSNPs, combined with the rigid nanoparticle core, contributes to the observed slow-release kinetics. The encapsulation of LL37 within CSNPs presents a promising strategy to enhance its therapeutic potential by ensuring a sustained release and improved stability. The observed release kinetics are consistent with the previous literature and further validate the applicability of a first-order release model for predicting CSNP-based drug delivery systems.

### 3.4. Biocompatibility of CSNPs

**Figure 4:**
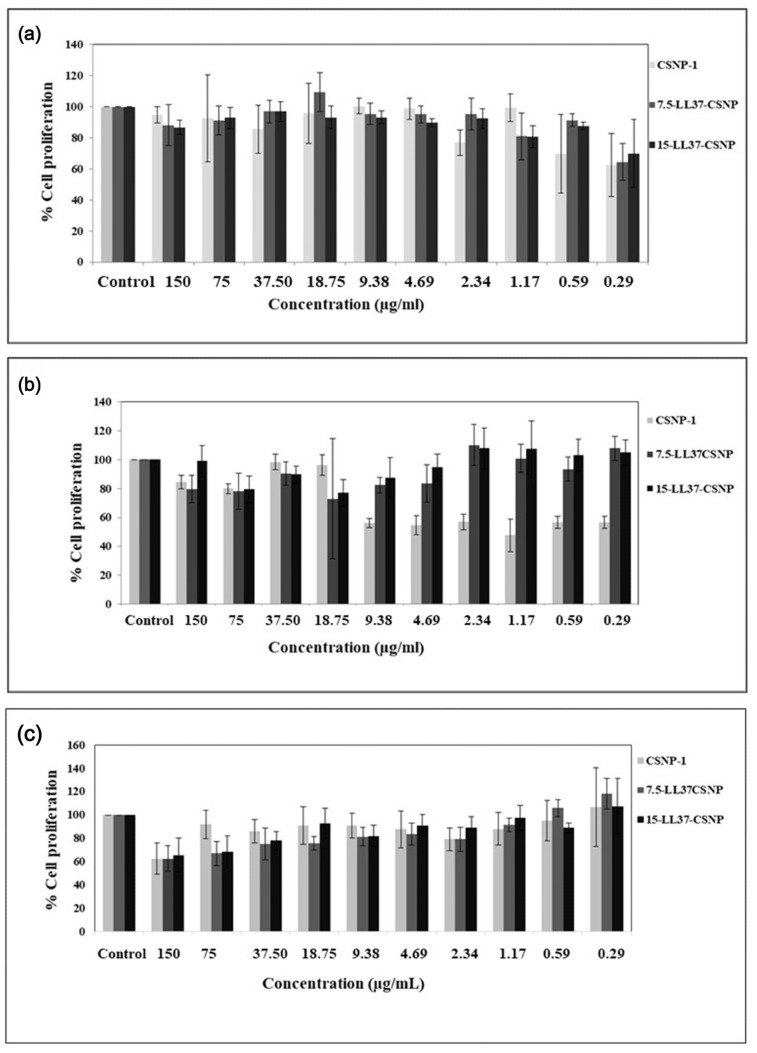
Effects of CSNP formulations on cell proliferations: (**a**) CSNP-1, 7.5-LL37-CSNP, and 15-LL37-CSNP at 24 h; (**b**) CSNP-1, 7.5-LL37-CSNP, and 15-LL37-CSNP at 48 h; and (**c**) CSNP-1, 7.5-LL37-CSNP, and 15-LL37-CSNP at 72 h.

An in vitro cell proliferation assay was conducted to assess the cytotoxic effect of CSNP-1, 7.5-LL37-CSNP, and 15-LL37-CSNP on keratinocyte cells. The cell proliferation graphs for the CSNP-1, 15-LL37-CSNP, and 7.5-LL37-CSNP groups at 24, 48, and 72 h are presented in Figure 4. The results demonstrate that there was no significant decrease in the cell viability over a concentration range from 150 µg/mL to 0.29 µg/mL for the CSNP-1, 7.5-LL37-CSNPs, and 15-LL37-CSNPs groups at 24, 48, and 72 h (Figure 4). The cell viability exceeding 70% representing the control cell viability can be interpreted as an indication of the suitability of the CSNP groups in facilitating cell proliferation according to the ISO10993-5 standard [64]. The cell proliferation percentages of the CSNP-1, as well as the 7.5-LL37-CSNPs and 15-LL37-CSNPs groups, across 10 different concentrations exceeded 70%, indicating biocompatibility. Similarly, Costa et al. [65] stated that, when compared with control conditions, all tested concentrations of CSNPs were found to be biocompatible with keratinocyte cells, indicating their minimal effect on cellular metabolism. In another study, Piras et al. [56] highlighted the excellent role of loading free temporin onto CS-NPs in reducing the toxicity profile in mammalian cells during cytocompatibility assessment. Similarly, according to the study by Fahimirad et al., the cytotoxic effects of CSLL37NPs on human dermal fibroblast (HDF) cells were evaluated using an MTT assay. The study reported that neither the CSLL37NPs nor the blank CSNPs (at concentrations up to 4096 μg/mL) caused a significant reduction in cell viability. In contrast, a notable decrease in cell viability was observed only in cells treated with free LL37 at concentrations higher than 64 μg/mL [55].

The human keratinocyte cell line was selected for the in vitro biocompatibility evaluation of both blank and LL37-loaded CSNP formulations due to its direct relevance to epidermal tissue. Keratinocytes are the predominant cell type in the outermost layer of the skin and play a key role in the wound healing process. The primary goal of this study was to assess the cytotoxic effects of the developed nanoparticle formulations as a preliminary step toward their potential application in future three-dimensional (3D) epidermal tissue models for wound healing therapy. Using keratinocyte cells in a two-dimensional (2D) culture system allows for a controlled and reproducible environment to evaluate hte potential cytotoxicity and effects on cell proliferation. This provides a fundamental understanding of the interaction between the CSNP formulations and skin-representative cells. Ultimately, the findings serve as an essential preclinical screening to determine the suitability of these delivery systems for advanced tissue-engineered skin models and therapeutic applications in wound healing.

**Figure 5:**
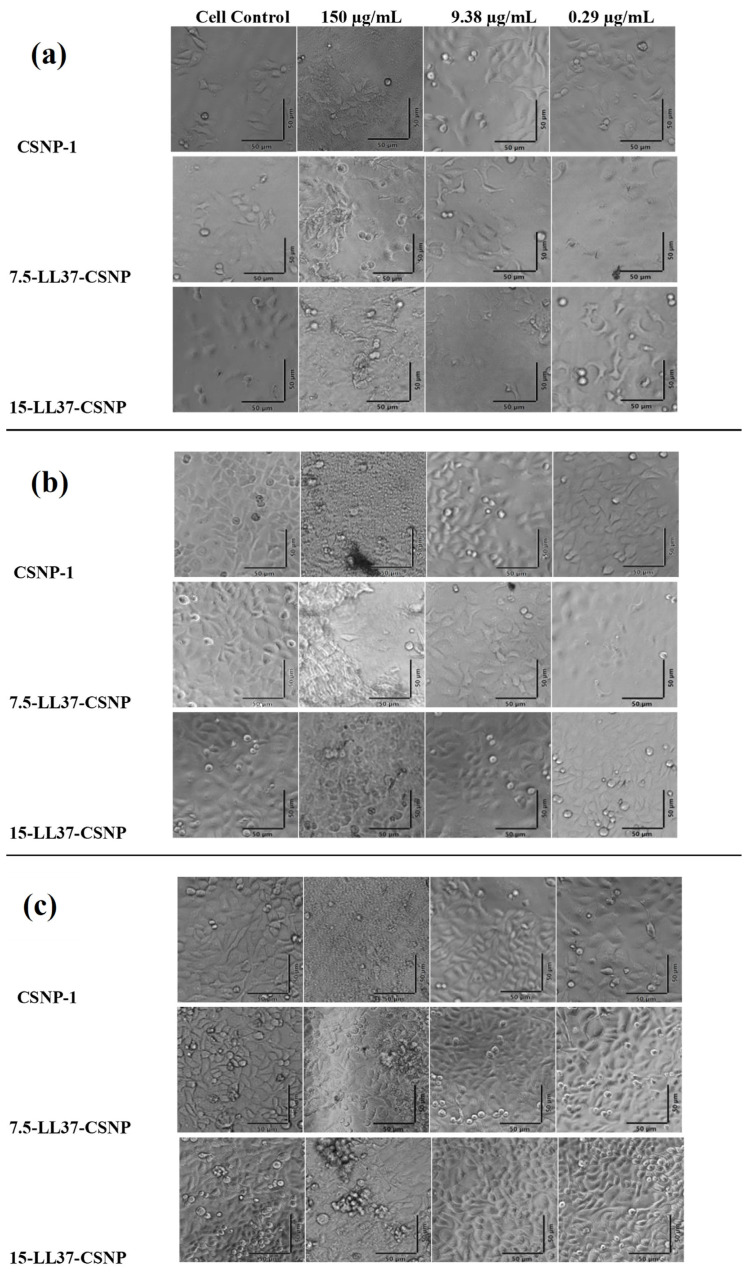
Microscope images of CSNP-1, 7.5-LL37-CSNP, and 15-LL37-CSNP after (**a**) 24, (**b**) 48, and (**c**) 72 h according to control and treatment groups.

Microscopic images corresponding to CSNP-1, 7.5-LL37-CSNP, and 15-LL37-CSNP at 24, 48, and 72 h (from left to right) are presented in Figure 5, in comparison to the control group and across different dosages.

Across all groups and time points, keratinocyte cells retained a normal epithelial morphology, with intact membranes, a homogeneous distribution, and no visual indicators of cytotoxicity, such as cell shrinkage, detachment, or lysis. The observed cytocompatibility is supported by a recent comprehensive review by Frigaard et al. (2022), which concluded that chitosan nanoparticles exhibit low cytotoxicity across various cell types and assays, and that new formulations should first undergo such a preliminary viability testing before in vivo application [66].

### 3.5. Antibacterial Activities of Blank and LL37-CSNPs

**Figure 6:**
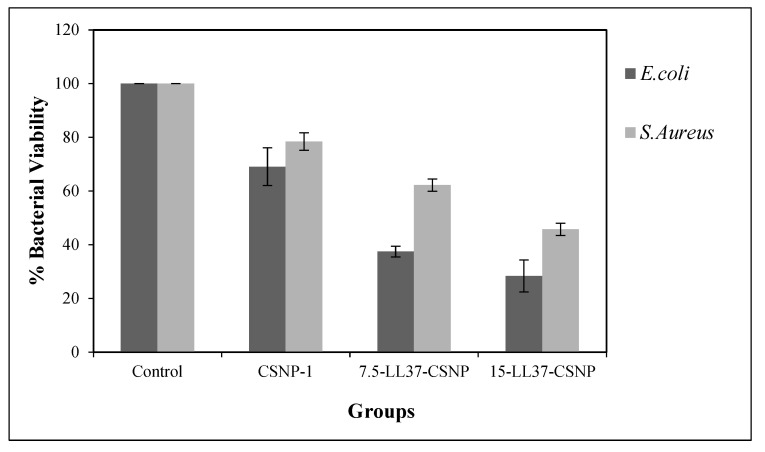
*E. coli* and *S. aureus* viability when treated with CSNP-1, 7.5-LL37-CSNPs, and 15-LL37-CSNPs.

The optimal experimental conditions were established to evaluate the antibacterial efficacy of CSNP-1, 15-LL37-CSNP, and 7.5-LL37-CSNP formulations against *E. coli* and *S. aureus* bacterial strains over a 16 h incubation period. A comparative analysis of CSNP-1, 7.5-LL37-CSNP, and 15-LL37-CSNP, as illustrated in Figure 6, demonstrated that both formulations significantly reduced the bacterial viability. Notably, the incorporation of LL37 into CSNPs enhanced the bactericidal activity relative to the blank CSNPs, with a more pronounced effect observed against *E. coli*. The antimicrobial efficacy of blank CSNPs is attributed to the intrinsic properties of chitosan, which, in combination with the nanoscale size, provides a high surface area and enhanced interaction with bacterial cells [67,68]. However, despite the observed reduction in bacterial viability with CSNP-1, their antibacterial effect alone was insufficient to achieve the desired efficacy. Therefore, LL37 was incorporated into the CSNP-1 formulation, which exhibited optimal characteristics for enhanced antibacterial activity.

All of these properties make CS a strong and promising candidate for the encapsulation of antimicrobial agents as novel nanocarriers for the treatment of microbial infections. Chitosan has also been used as a polymeric vehicle for the delivery of bioactive peptides. Antimicrobial peptides (AMPs) encapsulated in CS nanocarriers have shown successful outcomes in animal models [69,70,71]. Based on previous reports of in vivo wound healing studies, one of the major challenges associated with the application of free LL37 is the imbalance and rapid degradation at the wound site. Therefore, the desired therapeutic effect requires higher dosages and more frequent administrations. However, the encapsulation of peptides protects them from harsh environmental conditions, thereby reducing their susceptibility to degradation [59,60]. The encapsulation of LL37 within CSNPs can be considered a strategy for enhancing the stability of the AMP. Similarly, Yu et al. (2021) demonstrated that conjugating the antimicrobial peptide microcin J25 (MccJ25) to chitosan nanoparticles resulted in improved stability. In vivo studies have shown that CSLL37NPs lead to more effective healing outcomes and exhibit a superior antibacterial performance under in vivo conditions [72]. Another study confirmed that carboxymethyl chitosan nanoparticles loaded with the bioactive peptide OH-CATH30 significantly accelerated wound healing compared to free OH30 or CMCSNPs [73].

Previous studies have demonstrated the potent antimicrobial properties of LL37. For instance, Noore et al. [74] reported that LL37 rapidly eradicates both extracellular and intracellular *S. aureus* compared to conventional antibiotics. Furthermore, Neshani et al. [20] highlighted LL37’s broad-spectrum antimicrobial activity, showing lethal effects against *Acinetobacter baumannii*, *Staphylococcus aureus*, *Pseudomonas aeruginosa*, *enterococci*, and *Escherichia coli*. The findings of this study align with the existing literature, suggesting that the antimicrobial mechanisms of LL37-loaded CSNPs extend to both Gram-positive and Gram-negative bacterial strains.

Comparable studies have also explored the efficacy of chitosan-based nanoparticles for antibacterial applications. Piras et al. [56] reported a 4-log reduction in *Staphylococcus epidermidis* bacterial counts using temporin-loaded CS-NPs over a four-day period compared to CS-NPs alone. Similarly, Almaaytah et al. [75] demonstrated a 5-log reduction in *S. aureus* CFU/mL following treatment with antibacterial-short-peptide-loaded CS-NPs, confirming the potential of such nanoformulations in bacterial eradication.

## 4. Conclusions

This study provides valuable insights into the production and optimization of CSNPs, examining key parameters such as the chitosan and TPP concentrations, CS:TPP volume ratio, flow rate, pH, NaCl solution, Tween 80, sonication, and mixing time, all of which influence the particle size within the experimental groups. Notably, the addition of Tween 80 emerged as a critical factor in enhancing the nanoparticle stability and preventing aggregation, outperforming the NaCl solution in this regard within the CSNP (1-2-3-4) groups. The findings indicate that Tween 80 significantly impacts the average particle size, ZP, and size distribution indices of the CSNPs. In addition, the impact of Tween 80 and NaCl on the physicochemical characteristics of the nanoparticles—such as the particle size, polydispersity index (PDI), and surface charge—was examined during the ionic gelation process. Given that these additives can significantly affect the nanoparticle stability and morphology, this aspect represents an important technical consideration that has received limited attention in previous studies.

Utilizing the optimal formulation parameters from the CSNP-1 group, LL37-loaded CSNPs were successfully synthesized at concentrations of 30, 15, and 7.5 µg/mL. An encapsulation efficiency analysis revealed that the 7.5-LL37-CSNP and 15-LL37-CSNP samples achieved a satisfactory efficiency, making them suitable candidates for further experimental evaluations. This study systematically investigated the relationship between the encapsulation efficiency and particle loading capacity by conducting loading experiments at varying LL37 concentrations, ultimately identifying the optimal concentration. Thus, the study offers a unique contribution to the often-neglected issue of loading optimization in the current literature. While the CSNP-1 group demonstrated inherent antimicrobial properties, these effects alone were insufficient to achieve the desired level of efficacy. However, the incorporation of LL37 into the CSNPs significantly enhanced their antimicrobial potential, with the 15-LL37-CSNP group exhibiting superior efficacy compared to the 7.5-LL37-CSNP group. This synergistic combination effectively strengthens the antimicrobial action, rendering the formulation highly effective.

Importantly, the biocompatibility assessment confirmed that both the blank CSNP-1 and the LL37-loaded formulations (7.5-LL37-CSNP and 15-LL37-CSNP) did not exert cytotoxic effects on keratinocyte cells. This finding, coupled with the successful optimization of nanoparticle sizing, underscores the potential of these formulations for advanced biomedical applications. In conclusion, building upon the findings of this study, future research may focus on evaluating the functional performance of LL37-loaded CSNPs in advanced three-dimensional (3D) in vitro dermal models that better recapitulate the structural and biological complexity of human skin. Such investigations could provide critical insights into the cellular responses, tissue integration, and the controlled release behaviour of the system under physiologically relevant conditions. Additionally, the incorporation of targeting ligands or stimuli-responsive elements could be explored to further enhance site-specific delivery and therapeutic precision. From a translational standpoint, assessing the long-term physicochemical stability, scalability of the production process, and storage conditions will be essential steps toward clinical applicability. The adaptable nature of the chitosan-based nanoparticle platform also opens avenues for its extension to other therapeutic agents, including anti-inflammatory peptides, growth factors, or nucleic-acid-based drugs. Collectively, these directions underscore the broader potential of this delivery system not only for antimicrobial applications, but also for future use in regenerative medicine, tissue engineering, and personalized therapeutics.

## Figures and Tables

**Table 1:** The parameters utilized for blank CSNP production.

Groups	CS (mg/mL, pH 5)	TPP (mg/mL, pH 2)	CS/TPP (*v*/*v*)	NaCI (mM)	Tween80
CSNP-1	0.7	0.3	2:1	-	+
CSNP-2	0.7	0.3	2:1	100	-
CSNP-3	0.3	0.1	2:1	-	+
CSNP-4	0.3	0.1	2:1	100	-

**Table 2:** The average particle size, PDI, and ZP values of the groups.

Groups	Mean Particle Size (nm)	PDI	ZP (mV)
CSNP-1	180.40 ± 2.16	0.289 ± 0.00	40.57 ± 1.82
CSNP-2	198.00 ± 4.48	0.259 ± 0.03	43.68 ± 1.40
CSNP-3	291.90 ± 6.65	0.364 ± 0.01	46.26 ± 0.72
CSNP-4	496.40 ± 9.80	0.321 ± 0.00	35.75 ± 0.97
7.5-LL37-CSNP	195.60 ± 3.50	0.257 ± 0.02	19.26 ± 1.39
15-LL37-CSNP	210.90 ± 2.59	0.306 ± 0.02	51.21 ± 0.93

**Table 3:** % EE based on the initial LL37 concentration.

LL37 (µg/mL)	EE (%)
30 µg/mL	55.57 ± 11.90
15 µg/mL	80.32 ± 4.94
7.5 µg/mL	97.81 ± 2.72

**Table 4:** R^2^ values for the 24-day release kinetics of 7.5-LL37-CSNP and 15-LL37-CSNP formulations across various models.

	7.5-LL37-CSNP					15-LL37-CSNP				
Release Kinetics Model	R^2^	RMSE	RSS	AIC	BIC	R^2^	RMSE	RSS	AIC	BIC
First-Order Kinetics	0.9771	2.8708	197.7961	54.6204	56.9765	0.9724	5.3215	679.6389	84.2442	86.6003
Korsmeyer–Peppas	0.9680	3.3915	276.0563	62.6212	64.9773	0.9616	6.2766	945.5007	92.1679	94.5240
Zero-Order Kinetics	0.9569	3.9350	371.6292	69.7562	72.1123	0.9518	7.0313	1186.5542	97.6181	99.9742
Hixson–Crowell	0.8958	6.1189	898.5776	90.9462	93.3023	0.8744	11.3493	3091.3721	120.5996	122.9557
Higuchi	0.8760	6.6747	1069.2336	93.1194	94.2975	0.8454	12.5909	3804.7675	123.5830	124.7610

## Data Availability

The data are contained within the article.
